# Double Threaded Screw Fixation for Bilateral Stress Fracture of the Medial Malleolus

**DOI:** 10.1155/2014/729035

**Published:** 2014-01-28

**Authors:** Ryo Kanto, Shigeo Fukunishi, Takatoshi Morooka, Daisuke Seino, Takayuki Takashima, Shinichi Yoshiya, Juichi Tanaka

**Affiliations:** ^1^Department of Orthopaedic Surgery, Hyogo College of Medicine, 1-1 Mukogawa-cho, Nishinomiya, Hyogo 663-8501, Japan; ^2^Takashima Clinic, 1-13-22 Sawaraginishi, Ibaraki, Osaka 567-0868, Japan

## Abstract

An 18-year-old college basketball player presented with continued ankle pain. A radiographic examination showed bilateral medial malleolus stress fractures. Considering the prolonged history and refractory nature of this injury, surgery was adopted as a treatment option. At surgery, the fracture site was percutaneously fixed using two cannulated double threaded screws. Surgery for each side was sequentially performed two months apart. Prompt bony healing was attained after surgery, and the patient could return to his previous sports level six months after the first surgery without subsequent recurrence.

## 1. Introduction 

Repetitive loading during regular strenuous sports activity may cause stress fractures necessitating interruption of play. Its incidence in athletes has been reported to be around 2.0% [1–3]. The most common location is on the posteromedial-concave side of the tibial shaft [1], while stress fractures of medial malleolus are extremely rare. Shelbourne described 6 patients with stress fracture of this type in 1988 [4]. All of the patients were involved in running or jumping activities such as basketball, long distance running, and football. The typical clinical sequence of this stress fracture is gradual onset of pain and discomfort over the medial malleolus followed by prolonged symptoms. The radiological appearance is characterized by a vertically oriented fissure originating from the tibial plafond and medial malleolus junction or an obliquely arched radiolucent line through the medial malleolus; however, routine radiographs often appear normal at initial presentation. Therefore, for patients with clinical features suggestive of this stress fracture, bone scan or MRI may be considered for early diagnosis [5–7].

Since this fracture is mostly encountered among high level athletes, prompt diagnosis with aggressive intervention is critical to enable early return to original sports activity. The basic treatment option for stress fractures, in general, is conservative measure consisting of cessation of running and jumping activities; however, stress fracture of the medial malleolus is often complicated with delayed healing or recurrence necessitating surgery.

In this case report, we present a case of a college basketball player, who sustained bilateral medial malleolus stress fractures and sequentially underwent surgical treatment using double threaded screws. He was able to successfully go back to the original sports activity following the bilateral surgeries. In previous literatures, bilateral medial malleolus stress fracture has been reported in only one paper (in Germany) by Steckel et al. [8].

## 2. A Case Report

An 18-year-old college basketball player presented with discomfort and pain in his left ankle. He noted the symptoms approximately a month before his initial visit to our hospital without a history of preceding ankle injury. He had played basketball during his junior high and high school period and just started his career with the first season of the Division I college basketball league.

**Figure 1:**
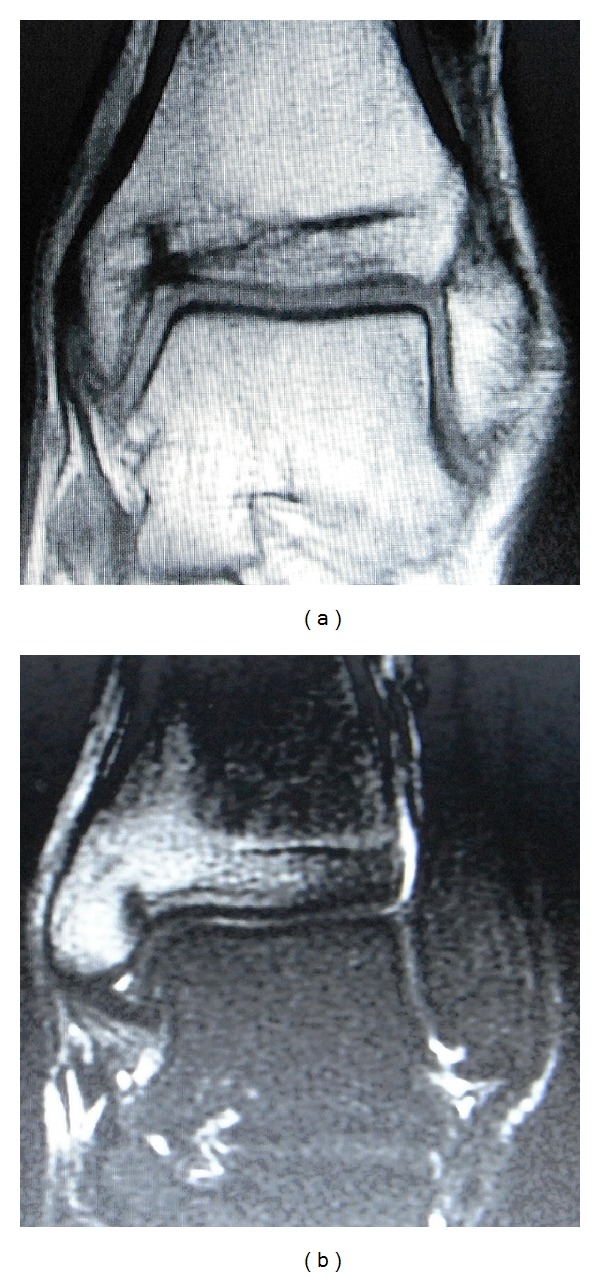
(a) T1-weighted MR image demonstrates a vertical linear lesion of low signal intensity originating from the superomedial corner of the ankle. (b) STIR (short T1 inversion recovery) image reveals bone marrow edema surrounding the linear lesion.

Physical examination on his initial presentation revealed mild swelling and localized tenderness over the anteromedial aspect of the ankle with mild limitation of dorsiflexion. Although a plain radiograph did not reveal any abnormality, a T1-weighted coronal MR image demonstrated a vertical linear lesion of low signal intensity originating from the junction between the tibial plafond and the medial malleolus (Figure 1(a)) and an STIR (short T1 inversion recovery) image revealed a bone marrow edema surrounding the linear lesion.

**Figure 2:**
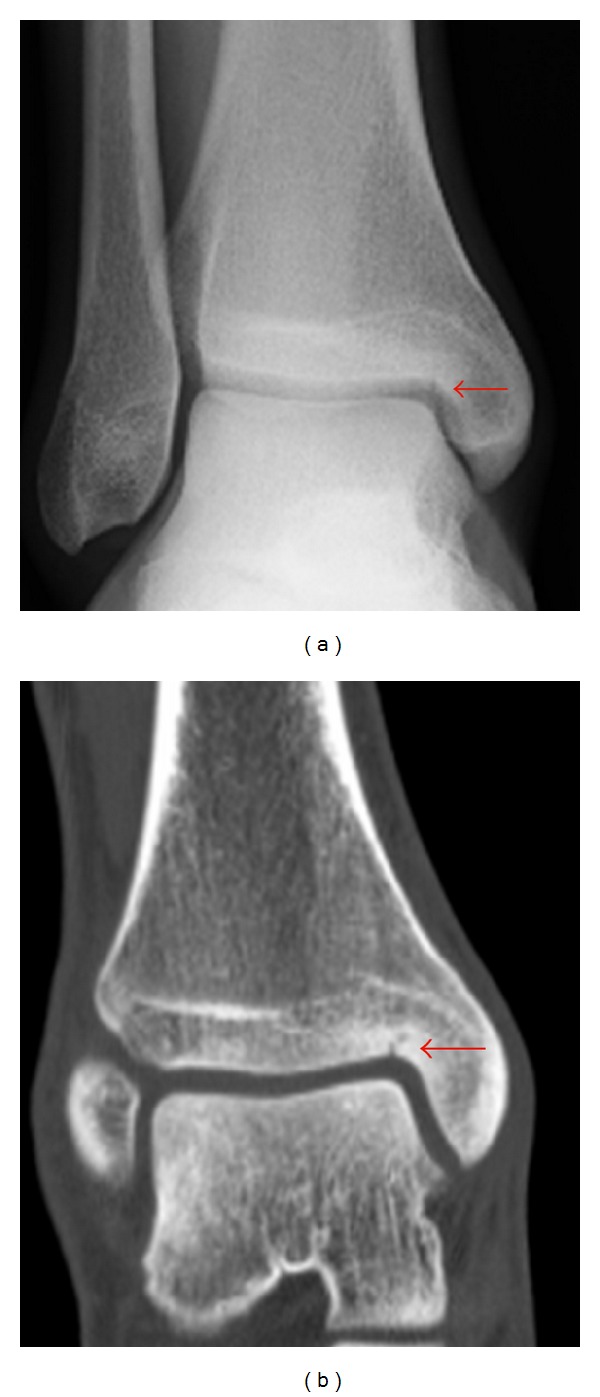
Anteroposterior radiograph (a) and coronal CT image (b) of the right ankle reveal a linear fracture line originating from superomedial corner of the ankle (arrow).

Based on these clinical and image findings, a diagnosis of stress fracture of the medial malleolus was made. In order to evaluate the fine bony structure, a CT examination was performed for both ankles. A linear radiolucent line corresponding to the MRI finding was identified in the left ankle, while a similar lesion was incidentally found in the contralateral asymptomatic right ankle (Figure 2(a)). The patient did not recall any injury or symptoms on this side. A radiograph of the right ankle showed a linear fracture line originating from superomedial corner of the ankle (Figure 2(b)).

**Figure 3:**
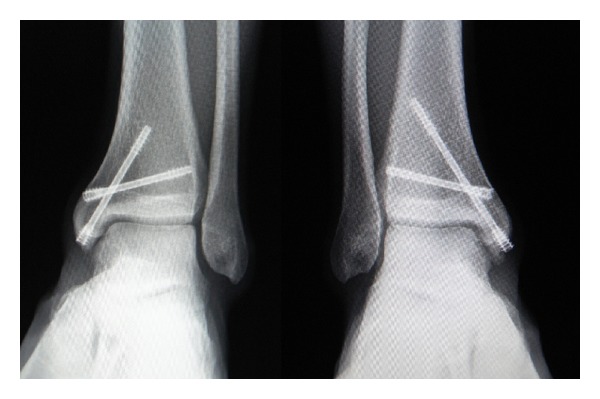
Anteroposterior radiograph at 4 years after surgery shows continued and complete bony healing in both ankles.

Considering the clinical features of this stress fracture characterized by delayed healing with prolonged morbidity, surgical intervention was indicated for the left ankle lesion. At surgery, the fracture site was percutaneously fixed using two cannulated double threaded screws (double thread screw Japan, Meira, Nagoya, Japan) (Figure 3). Postoperatively, immobilization with a short leg cast was applied and no-weight bearing was permitted for two weeks. Subsequently, partial weight bearing and range of motion exercises were initiated with the use of the Air Cast Brace. Six weeks after surgery, the patient began jogging after radiological confirmation of healing. Thereafter, he complained of discomfort in the contralateral right ankle. Repeat CT examination revealed a clear vertical fracture line originating from the superomedial corner of the right ankle. The fracture line appeared expanded compared to the previous CT image taken two months ago. Considering the progressive nature of this lesion, surgery with identical technique to the left side was performed for the stress fracture on the right side two months after the initial surgery. The postoperative regimen followed the protocol adopted for the left side. Subsequently, uneventful bony healing was attained and he could return to competitive level basketball without any complaints for either ankle six months following the initial surgery of the left side. Finally, he could complete the fourth regular season of the Division I college basketball league without recurrence (Figure 3).

## 3. Discussion 

Stress fracture of the medial malleolus is relatively rare and has been reported sporadically. Amongst the reported cases, bilateral involvement is especially rare and only one case report has been identified in literatures [8]. The stress fracture of this type is known to pose difficulties in both diagnosis and treatment.

Regarding the diagnostic criteria for this lesion, Shelbourne listed the characteristic clinical features as follows: (1) tenderness over the medial malleolus with joint effusion, (2) pain during activity before an acute episode, and (3) a vertical fracture line from the tibia plafond on radiograph. However, a plain radiograph on initial presentation can be normal [4]. Therefore, for patients with suspicion of this stress fracture, MRI or bone scan are recommended for early detection of the bony lesion. In this reported case, the initial radiograph taken one month following onset revealed no abnormality and diagnosis was confirmed by MRI. Additionally, the lesion on the contralateral side was identified incidentally by a CT examination performed as part of a preoperative assessment of the symptomatic lesion on the other side. This clinical sequence showed the diagnostic difficulty for the stress fracture in this region.

Several factors that may predispose athletes to medial malleolus stress fractures have been reported in literatures. Schils addressed the influence of external factors including training errors, excessively hard training, and inadequate footwear [9]. By contrast, Orava focused on internal factors such as leg length discrepancy, forefoot varus, subtalar varus, pes cavus, and tibial varum [10]. No internal factors were identified in the reported case; however, an increase in practice intensity at the beginning of the first season of the college basketball league may have been a factor inducing excessive stress on the ankle associated with repetitive jumping and landing activities.

Since prolonged morbidity with delayed healing and recurrence is often encountered in the management course of this stress fracture, surgical intervention may be required especially for high level athletes. Shelbourne recommended open reduction and internal fixation for athletes with clear evidence of a fracture line on plain radiographs to promote early return to play [4, 5]. However, these authors recommended a conservative treatment option with casting for cases with negative radiographic findings. In Orava's case series, five of the eight patients were successfully managed with conservative treatment while surgery was required for three patients with fractures with displacement or delayed healing. A recent article by these authors (Orava et al.) in 2012 reported a clinical course of 10 patients who underwent surgery for this injury [11]. Among the 10 patients included in this case series, surgery was indicated for five patients after failed attempts of conservative treatment. Based on the review of previous literatures, Shabat et al. concluded that surgical treatment can be considered as a primary option for high level athletes [12]. In the present case, we sequentially operated on bilateral ankles with a period of two months in between. Since the right ankle was asymptomatic, we hesitated to adopt an immediate surgical option and surgery on this side was delayed. If bilateral surgeries were performed simultaneously for this patient, the time period for full return to play should have been shortened.

Regarding the method of surgical treatment, various procedures have been reported. Orava reported two cases with delayed union managed by drilling with a 2.2 mm drill bit [10], while Reider reported a patient with nonunion who underwent internal fixation with bone graft after debridement of fibrous tissues [13]. For patients presenting with short clinical history as the case reported here, screw fixation without bone graft has generally been adopted as a procedure option. Various types of screws such as 4.0 mm cancellous screws, 6.5 mm cannulated cancellous screws, and partially threaded 4.0 mm cancellous compression screws have been used in previous reports. In the present case, we performed percutaneous fixation using two cannulated double threaded screws. The screw of this type is commonly used for scaphoid fracture with favorable clinical results reported in literatures. Advantages of this screw include no prominence of the screw head and achievement of rigid fixation with compression applied to the fracture site. Additionally, percutaneous fixation may reduce surgical invasiveness inducing prompt postoperative functional recovery.

In conclusion, this case report examined the clinical course of a college basketball player with bilateral stress fracture of the medial malleolus who was successfully treated with percutaneous screw fixation. The use of cannulated double threaded screws in this situation was effective, and the patient could return to full sports activity six months after the first surgery and continued to play at the competitive level for the subsequent years without recurrence.
